# Modified Mason-Allen with Mattress Sutures and Double Row Suture Bridge in Musculotendinosis Rotator Cuff Tear

**DOI:** 10.1016/j.eats.2025.103541

**Published:** 2025-04-03

**Authors:** Renaldi Prasetia, Siti Zainab Bani Purwana, Greesea Dinamaria, Hermawan Nagar Rasyid, Agus Hadian Rahim

**Affiliations:** a Department of Orthopaedics – Traumatology, Universitas Padjadjaran, Hasan-Sadikin General Hospital, Bandung, Indonesia; b Faculty of Medicine, Universitas Padjadjaran, Hasan-Sadikin General Hospital, Bandung, Indonesia

## Abstract

Musculotendinous rotator cuff tear is rarely encountered, and the repair is challenging with high retear rate. There is no definitive guideline on how to manage the tear. We present step-by-step the modified Mason-Allen technique using horizontal mattress sutures and double-row suture bridge for musculotendinous rotator cuff tear repair, which is simple to implement and improves soft-tissue stability while avoiding tendon constriction.

Musculotendinous tear is rarely found in the rotator cuff.^1^^,^^2^ It is known to be located a few centimeters medial than the usual rotator cuff tears, often with intact tendon attachments, acute severe edema, and fatty infiltration.^1^^,^^3^ The rarity of the case could be attributable to the need of multifactorial causes such as various anatomic component contributions, intrinsic degeneration, and extrinsic factors.^1^ Secondary tear of the musculotendinous junction (MTJ) may occur, with 20% failure rate of primary rotator cuff repair.^3^

The procedure to treat the torn rotator cuff has developed from simple debridement to repair.^4^ Musculotendinous tear repairs aim to restore anatomic rotator cuff footprint, achieve adequate footprint decompression, minimize gap formation, and maximize ultimate load-to-failure.^5^ The repair techniques often restore only partial function^1^ Knot impingement on MTJ tissue, concentrated medial stress, or tissue strangulation also could lead to failure.^3^

The modified Mason-Allen technique, developed by Scheibel and Habermeyer, uses a complex single-row technique.^6^^,^^7^ It also has been described involving a double-row suture as well as a rip-stop consisting of horizontal mattress and simple sutures.^5^^,^^8^ The suture technique aims to provide high initial fixation strength while maintaining mechanical stability.^9^ We aim to outline step-by-step the modified Mason-Allen technique using horizontal mattress sutures and double-row suture bridge for musculotendinous rotator cuff tear repair. This study was approved by our institution’s ethical committee, and informed consent was provided by the patient.

## Surgical Technique

### Patient Positioning and Anesthesia

A regional interscalene block is administered before the induction of general anesthesia. The patient is positioned in a beach-chair position. The operative shoulder is prepared and draped with sterile techniques.

### Diagnostic Arthroscopy

We provide a video for this technique (Video 1). The midposterolateral portal acts as the viewing portal, where an arthroscope is inserted, whereas the anterior and posterior portals are used as the working portals. An additional midanterolateral portal is used for knot-tying.

### Subacromial Decompression, Bursectomy, Debridement, and Subscapularis Tendon Repair

The height of the subacromial space is opened and increased by performing subacromial decompression using a shaver (Incisor Plus Elite, 3.5 mm; Smith & Nephew) and a 90° tipped radiofrequency device (Ambient Super Turbovac; Smith & Nephew) to improve visualization.

### Supraspinatus Tendon Repair With a Modified Mason–Allen Technique

#### Step 1: Tissue Refreshment and Bone Decortication

The torn supraspinatus is debrided and mobilized to the original insertion. The bone is resurfaced by a shaver (Incisor Plus Elite, 3.5 mm; Smith & Nephew), with the cortex removed, for enthesis debridement on the supraspinatus foot print.

#### Step 2: Medial-Row Suture Anchor

**Fig 1:**
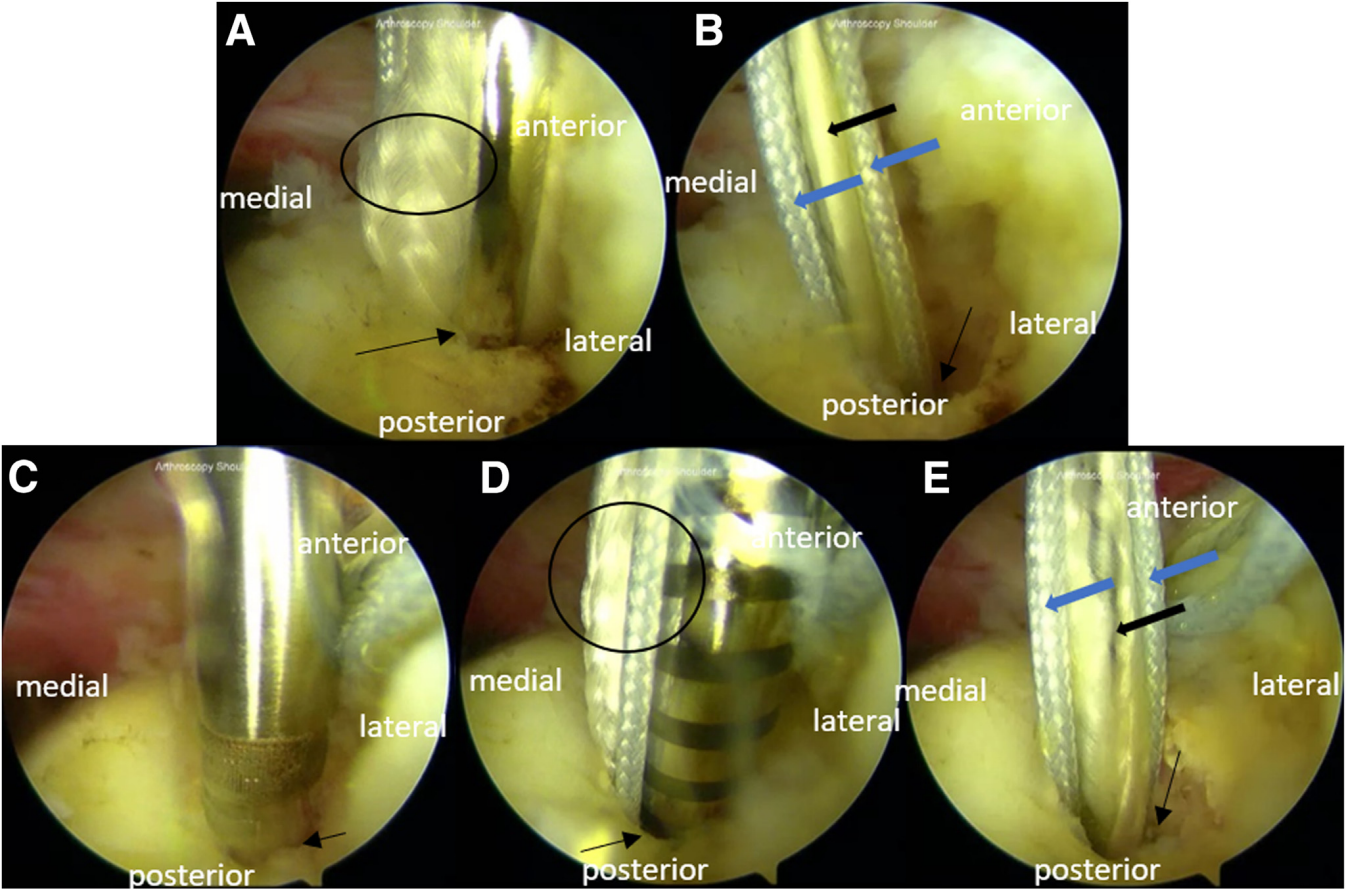
Medial-row anchor placement for the arthroscopic modified Mason-Allen technique for the repair of massive rotator cuff tear in the right shoulder with the patient positioned in the beach-chair position. The portals used are the midposterolateral (viewing), anterior (working), midanterolateral (working), and posterior (working). Medial anchors are located between the articular cartilage medially and the raw surface of the supraspinatus footprint laterally. (A) We perform anchoring with a 3.5 double-loaded anchor (Y-Knot RC, 2.8 mm; ConMed) after the intraosseous tunnel in the anterior part of the medial row was made. (B) We ensure proper fixation after the first medial row anchor is placed. (C) A second anchor (Y-Knot RC, 2.8 mm; ConMed) is placed in the intraosseous tunnel with 2- to 3-cm bridge distance posteriorly to the first anchor. (D) We insert the anchor and (E) ensure a proper fixation of the posterior medial-row anchor. Each of the double-loaded suture anchors consisted of one pair of tiger-colored threads and one pair of solid-colored threads. Arrow: anchor in the intraosseous tunnel; circle: double-loaded suture; blue thick arrow: solid-colored thread; and black thick arrow: tiger-colored thread.

The anchor insertion is taken care with a 45° angle. Anchors with solid- and tiger-colored threads are placed at the medial row in a pair of intraosseous tunnels posteriorly and anteriorly (Fig 1). The double-loaded suture anchors (Y-Knot RC, 2.8 mm; ConMed) are inserted in the medial row at the junction between the articular cartilage and the raw surface of the supraspinatus footprint from the anterior with a bridge distance of 2 to 3 cm.

#### Step 3: Horizontal Mattress Suture Management for Rip-Stop

**Fig 2:**
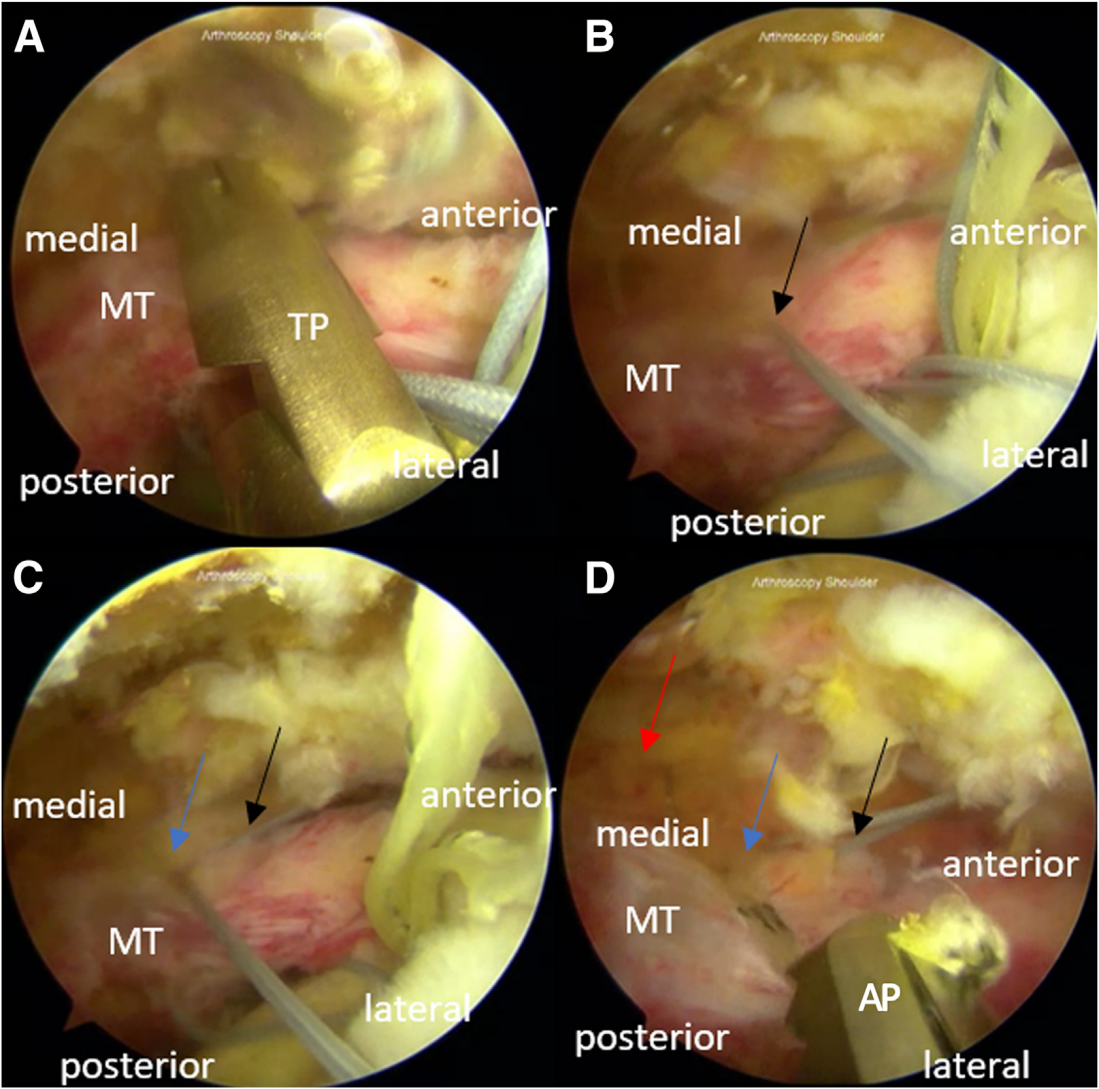
Thread passage from the medial-row anchors (articular side) to the bursal side of the torn rotator cuff for the arthroscopic modified Mason-Allen technique for the repair of massive rotator cuff tear in the right shoulder with the patient positioned in the beach-chair position. The portals used are the midposterolateral (viewing), anterior (working), midanterolateral (working), and posterior (working). (A-B) A solid-colored thread from the first medial row anchor is passed through the musculotendinous rotator cuff from the intra-articular side to the bursal side using a suture passer (TRUEPASS; Smith & Nephew), creating the first passage (hole A). (C) The second solid-colored thread is passed through the musculotendinous rotator cuff similarly, creating the second passage (hole B) 1 to 1.5 cm from hole A. (D) A pair of tiger-colored threads is penetrated through the third passage (hole C) using a suture passer (ACCU-PASS; Smith & Nephew) 0.5 to 1 cm medial from holes A and B from the intra-articular side to the bursal side. Black arrow: hole A; blue arrow: hole B; red arrow: hole C. (AP, ACCU-PASS; MT, musculotendinous; TP, TRUEPASS.)

A solid-colored thread from the anterior anchor is penetrated using a suture passer (TRUEPASS; Smith & Nephew), creating the first passage, from the intra-articular side to the bursal side (Fig 2). The first passage is placed anterior to the musculotendinous and 0.8 to 1.2 cm from the tear edge. The second solid-colored thread from the same anchor is then similarly passed through, creating the second passage, 1 to 1.5 cm posterior to the first passage. Penetration is made 0.5 to 1 cm medial to the first and second passages, taking both the tiger-colored threads from the first anchor through the third passage with the assistance of the suture passer (ACCU-PASS; Smith & Nephew).

**Fig 3:**
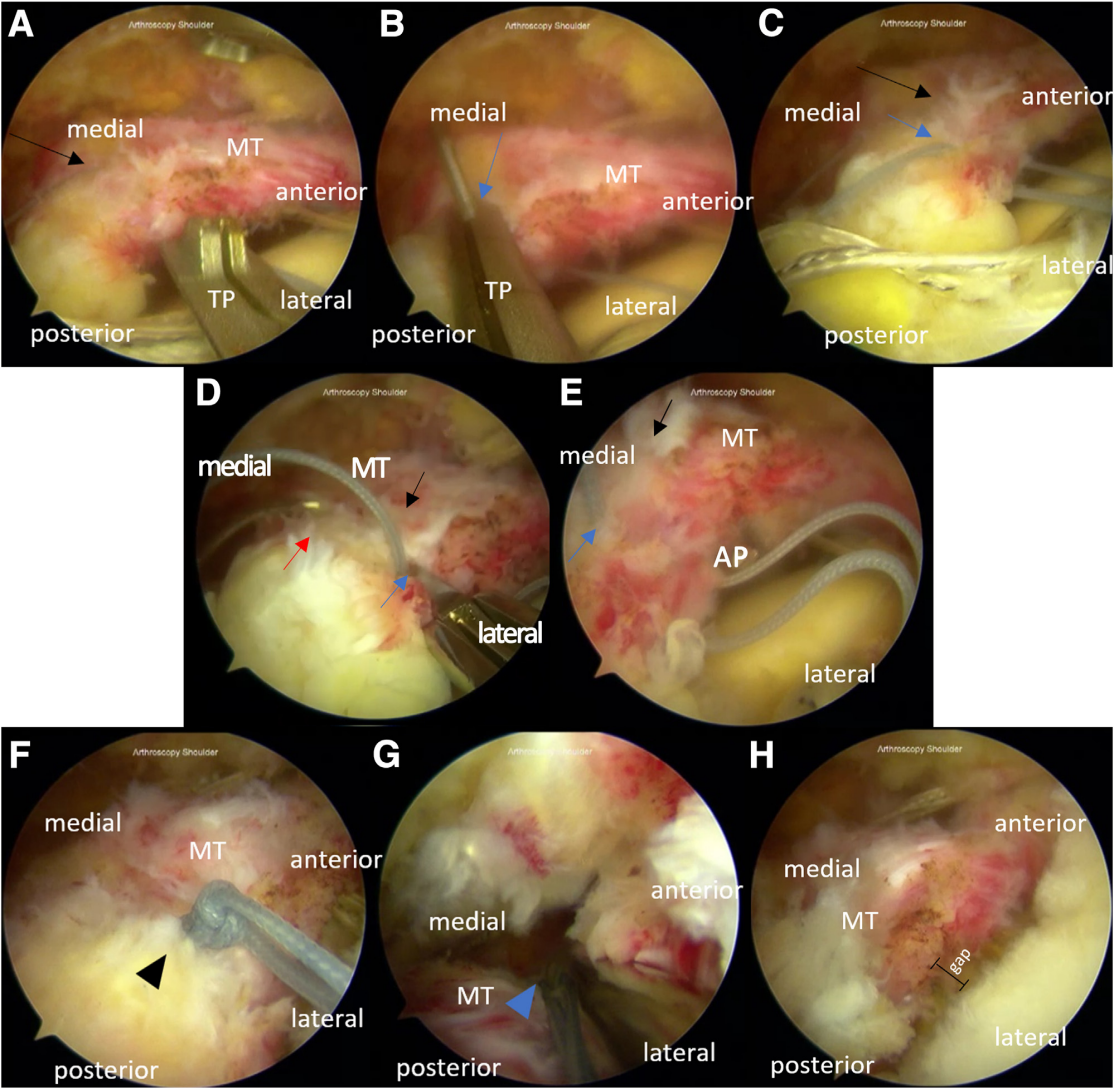
Horizontal mattress sutures and thread passage for the arthroscopic modified Mason-Allen technique for the repair of massive rotator cuff tear in the right shoulder with the patient positioned in the beach-chair position. The portals used are the midposterolateral (viewing), anterior (working), midanterolateral (working), and posterior (working). From the second anchor, (A) a solid-colored thread is passed through the musculotendinous rotator cuff 1 cm posterior from the second passage (hole B), similar to the solid-colored thread from the first anchor, creating the fourth passage (hole D). (B-C) The fifth passage (hole E) is created by passing through the remaining solid-colored thread from the second anchor using a suture passer (TRUEPASS; Smith & Nephew) 1 cm from hole D. (D, E) A suture passer (ACCU-PASS; Smith & Nephew) is used to penetrate the pair of tiger-colored threads from the second anchor, creating the sixth passage (hole F). (F) Solid threads coming out of holes D and E are knotted. (G) Knot tying is done to the threads coming out of holes A and B. With all the solid-colored threads knotted, we complete the rip-stop mattress suture. (F) The torn musculotendinous rotator cuff after the mattress suture is approximated to the footprint, but a gap between the 2 is still visible. Black arrow: hole D; blue arrow: hole E; red arrow: hole F; black triangle: posterior knot of the mattress suture; and blue triangle: anterior knot of the mattress suture. (AP, ACCU-PASS; TP, TRUEPASS.)

Solid color threads from the second and first anchor are passed similarly to the solid color thread from the first or anterior medial-row anchor, creating the fourth and fifth passages, which are placed 1 cm behind the second and third passage (Fig 3). The pair of tiger color thread in the second anchor is penetrated through the sixth passage, 0.5 to 1 cm medial to the fourth and fifth passage. Solid color sutures from the fourth and first passage are respectively tied with the sutures coming out of the fifth and second passage, creating the mattress suture.

#### Step 4: Double-Row Suture Bridge Technique

**Fig 4:**
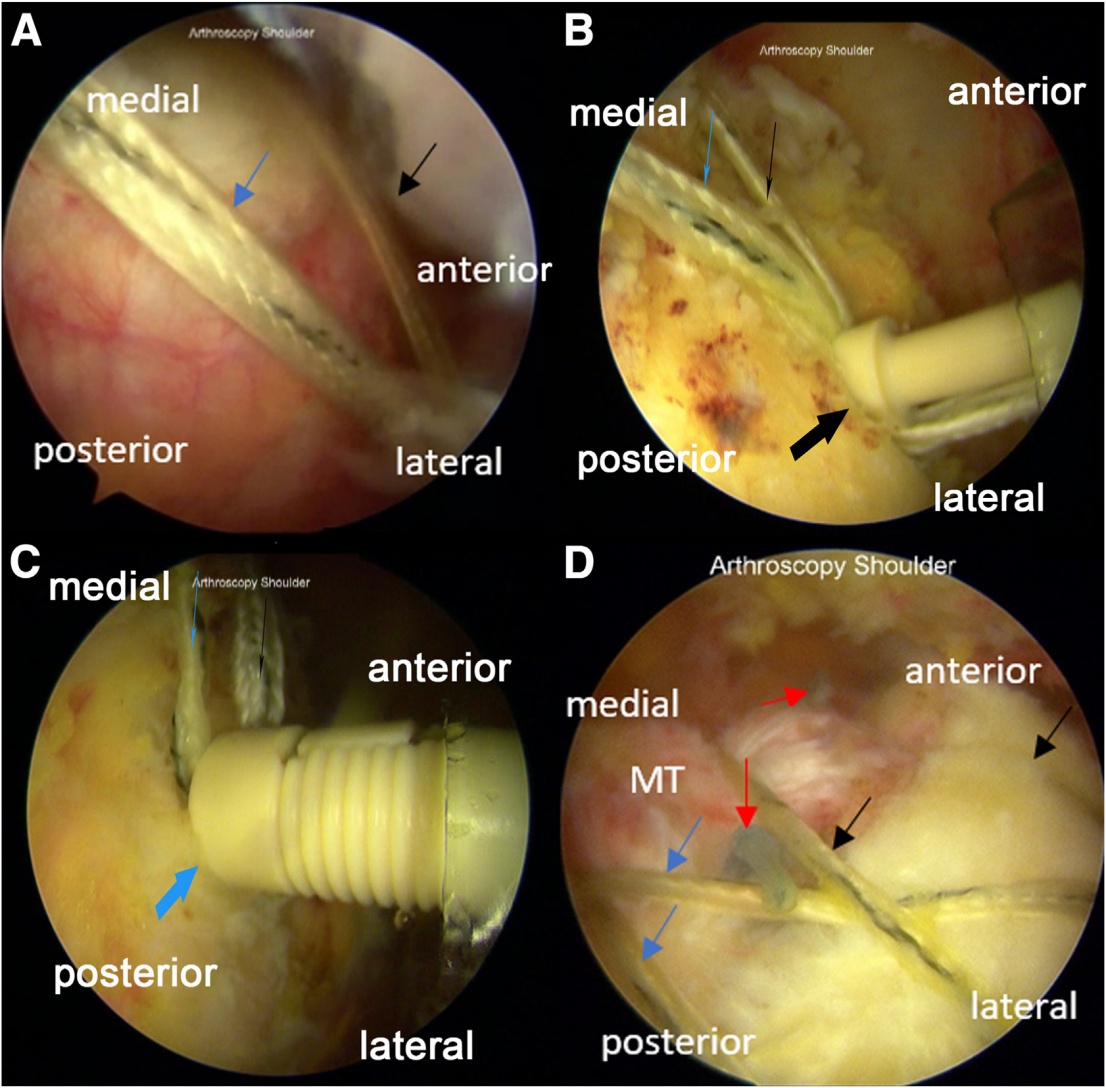
Double-row suture bridge for the arthroscopic modified Mason-Allen technique for the repair of massive rotator cuff tear in the right shoulder with the patient positioned in the beach-chair position. The portals used are the midposterolateral (viewing), anterior (working), midanterolateral (working), and posterior (working). (A) Tiger-colored threads from each of the first and second medial row anchors are brought to the border of the bicipitalis sulcus and (B) fixated as the anterior lateral-row anchor using a PopLok anchor (PopLok, 4.5 mm; ConMed). (C) The remaining tiger-colored threads from both the medial row anchors are then taken to the posterior side of the lateral row to be anchored (Multifix, 5.5 mm; Smith & Nephew) in front of the infraspinatus muscle. (E) A double-row suture bridge is created, removing the gap between the torn musculotendinous rotator cuff and the footprint. Black arrow: tiger-colored thread from hole C; blue arrow: tiger-colored thread from hole F; thick black arrow: anterior lateral row anchor; thick blue arrow: posterior lateral row anchor; and red arrow: knot of the mattress suture.

The 2 pairs of tiger threads are then fixated in the lateral row, with one end anchored in a crossing manner and the other anchored according to its anterior or posterior position, to the borders of the infraspinatus muscle and the sulcus bicipitalis (Fig 4). One thread from the third passage and one from the sixth passage are brought to the lateral row in front of the sulcus bicipitalis to be fixated with a knotless anchor (PopLok, 4.5 mm; ConMed). The remaining threads are taken to the front of the infraspinatus muscle and then similarly fixated with a knotless anchor (Multifix, 5.5 mm; Smith & Nephew). The knotless anchors once placed in the lateral row create the double-row suture bridge.

#### Step 5: Strength Reassessment

**Fig 5:**
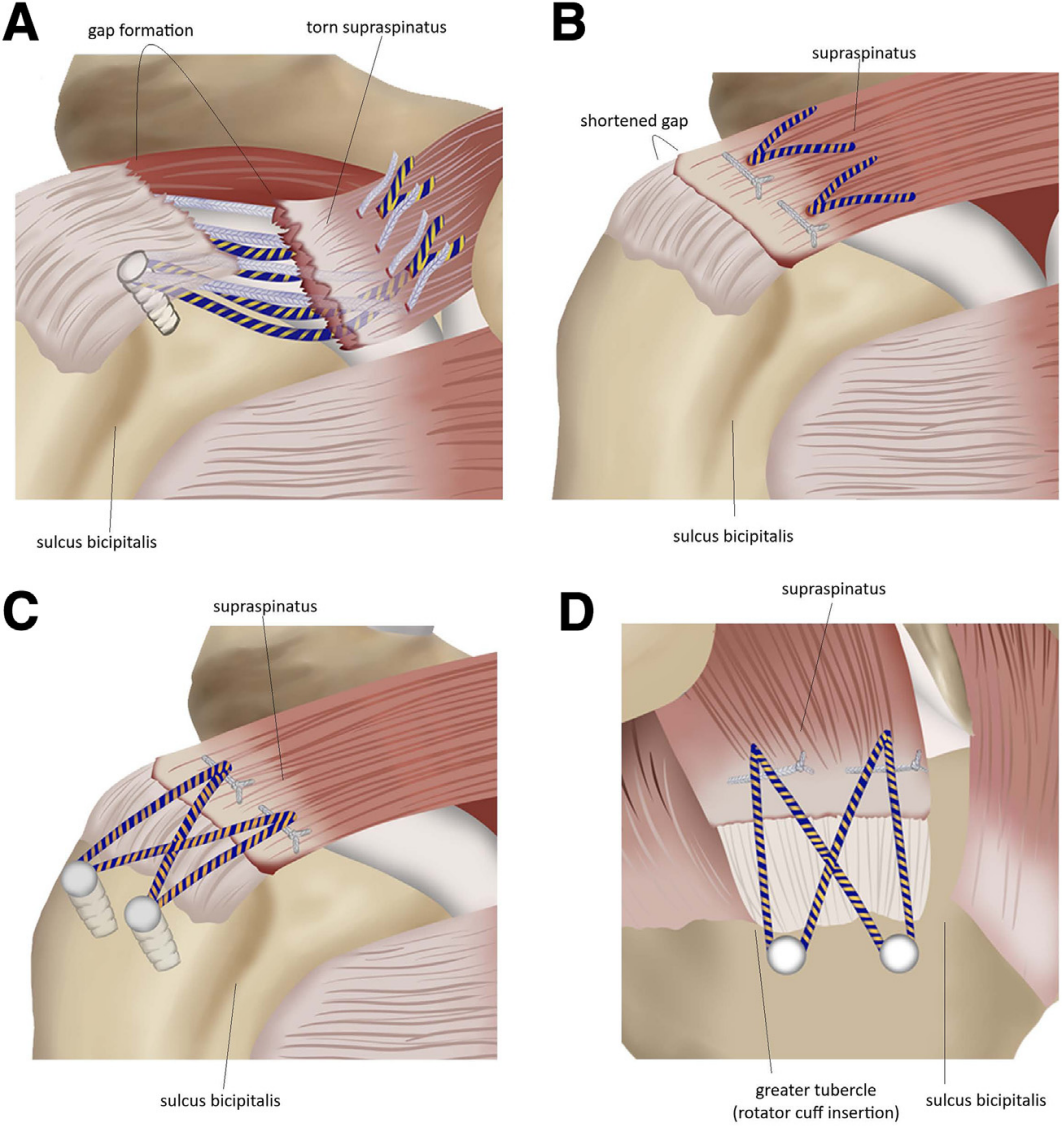
Illustrations of modified Mason-Allen technique using horizontal mattress suture and double-row suture technique for the repair of massive rotator cuff tear. (A) A pair of double-loaded sutures consisting of solid- and tiger-colored threads from the medial row anchors passing through the passages in the musculotendinous rotator cuff from the intra-articular side to the bursal side is shown. (B) Knotted mattress suture and (C) anchored double-row suture bridge. (D) Lateral view of the modified Mason–Allen technique with mattress sutures and double-row suture bridge.

The tied sutures are pulled, and the presence of a gap between the repaired musculotendinous cuff and the humeral head is arthroscopically inspected. It should be ensured that the sutures are not overtensioned. We illustrate the technique in Figure 5. The pearls and pitfalls of the steps in the procedure are described in Table 1.

**Table 1:** Pearls and Pitfalls of the Procedure

Surgical Steps	Pitfalls	Pearls
Patient positioning and anesthesia	Meticulous positioning and preparation are required	Less stress on the brachial plexus, better visualization of the glenohumeral and subacromial region, and great flexibility
	Paresthesia, often resolves on its own	Excellent analgesia and muscle relaxation
Diagnostic arthroscopy	Failure to identify associated lesions (depends on the surgeon)	Detailed assessment on the associated lesion in adjacent structures
Subacromial decompression and bursectomy	Neurovascular complication	Improved visualization
Tissue refreshment and bone decortication	Excessive abrasion	Bleeding allows proper healing
Medial row suture anchor	Soft-structured bone and insufficient area can cause an improper anchoring site	Removing gap between the tear and the footprint
Horizontal mattress suture management for rip-stop	Overtension can cause strangulation leading to ischemia	Provide attachments to the medial anchor and excellent rip-stop
Double-row suture bridge technique	Overtensioning the thread can lead to longitudinal tears. Soft-structured bone and insufficient area can cause an improper anchoring site	Removing gap between the tear and the footprint
Strength reassessment	Overtensioning can cause tear	Making sure the procedure is done properly, assuring proper attachment for healing

### Postoperative Rehabilitation

Immediate postoperative rehabilitation is conducted to maintain the passive range of motion. The affected shoulder is immobilized in the abduction brace with 30 to 45° abduction. The hand, wrist, and elbow active range of motion is maintained during this phase with pendulum and forward flexion exercises. Active shoulder motion exercises are allowed 6 weeks after the surgery. Normal activity should be regained 4 months after the procedure.

## Discussion

Repair of MTJ rotator cuff tear is challenging, with a high failure rate reported.^9^^,^^10^ Medially placed tears have a multifactorial etiology with a combination of acute or chronic trauma and subacromial impingement.^4^^,^^9^^,^^11^ Improvements of clinical results, such as pain, range of motion, strength, and function, are expected after repair.^12^ Arthroscopic repair is beneficial in reducing morbidity and pain.^6^ Ideally, it also provides a strong fixation, consequently promoting healing and allowing a more vigorous process of rehabilitation exercises.^13^ Various suture techniques are applicable for the arthroscopic rotator cuff repair.^14^

Overtension in rotator cuff repair could cause compression that could result in strangulation and, eventually, necrosis. The suture bridge serves as an attempt to reduce this stress concentration. It also provides a better footprint coverage and an area of pressurized contact, ensuring no gap formation. The double-row and suture bridge had a lesser rate of retear compared with the single-row suture; however, a more medial tension-bearing row, stronger suture material than the impaired tissue, and oblique passage of instruments would put tension and create openings that could lead to retear.^15^ Horizontal mattress sutures perpendicular to the approximating sutures, as seen in the Mason-Allen stitch technique, had been used for rip-stop to prevent cut-through.^16^

The most important step in rotator cuff repair is the proper reattachment of the ruptured rotator cuff tendons to the bony bed.^5^ The Mason-Allen suture has a very strong tissue-holding property. The technique has been modified with claims that the modified Mason–Allen suturing technique provides the greatest pull-out strength and the least potential of soft-tissue injury compared with other stitches used in rotator cuff repairs.^5^^,^^17^^,^^18^ As used in the modified Mason-Allen suture, suture anchors are shown in the literature to be superior to other fixation devices without suture.^5^ Lee et al.^18^ stated that the modified Mason-Allen suture with a combination of mattress and single suture offers a constant and balanced contact pressure of the tendon to the bony bed. Suture techniques used must ensure mechanical stability until the healed tissue has established osteofibroblastic integration.^7^

Although this technique has not been examined histologically, a clinical study in a similar modified Mason-Allen technique did not show any aseptic tendon necrosis. Tendon strangulation also needs to be kept to a minimum to avoid local metabolic compromise and damage addition to the reattached tendon.^7^ The modified Mason-Allen has the advantages of reduced rotator cuff tendon strangulation, impingement, and irritation that may be caused by the knot.^18^

## Disclosures

All authors (R.P., S.Z.B.P., G.D., H.N.R., A.H.R.) declare that they have no known competing financial interests or personal relationships that could have appeared to influence the work reported in this paper.

## Supplementary Data

Video 1 Step-by-step procedure of arthroscopic modified Mason-Allen technique using mattress sutures and double-row suture technique for the repair of massive rotator cuff tear in the right shoulder with patient positioned in beach-chair position. The portals used were midposterolateral (viewing), anterior (working), midanterolateral (working), and posterior (working).

## References

[1] Taneja A.K., Kattapuram S.V., Chang C.Y., Simeone F.J., Bredella M.A., Torriani M. (2014). MRI findings of rotator cuff myotendinous junction injury. AJR Am J Roentgenol.

[2] Miranda M.O., Bureau N.J. (2019). Supraspinatus myotendinous junction injuries: MRI findings and prevalence. AJR Am J Roentgenol.

[3] Hall T., Danielson K., Brandenburg S., Matelic T. (2020). A case series of recurrent myotendinous rotator cuff tears repaired and augmented with dermal allograft: Clinical outcomes at two years. J Shoulder Elbow Surg.

[4] Lashgari C., Redziniak D. (2012). The natural history of rotator cuff tears. Curr Orthop Pract.

[5] Lee K.W., Yang D.S., Lee G.S., Ma C.H., Choy W.S. (2018). Clinical outcomes and repair integrity after arthroscopic full-thickness rotator cuff repair: Suture-bridge versus double-row modified Mason-Allen technique. J Shoulder Elbow Surg.

[6] Khalil M.H., Rashwan A.S. (2018). Arthroscopic rotator cuff repair using modified Mason-Allen versus double row suture bridge techniques. Egypt Orthop J.

[7] Scheibel M.T., Habermeyer P. (2003). A modified Mason-Allen technique for rotator cuff repair using suture anchors. Arthroscopy.

[8] Noyes M.P., Lederman E., Adams C.R., Denard P.J. (2018). Triple-loaded suture anchors versus a knotless rip stop construct in a single-row rotator cuff repair model. Arthroscopy.

[9] Millett P.J., Hussain Z.B., Fritz E.M., Warth R.J., Katthagen J.C., Pogorzelski J. (2017). Rotator cuff tears at the musculotendinous junction: Classification and surgical options for repair and reconstruction. Arthrosc Tech.

[10] Gyftopoulos S., Beltran L.S., Gibbs K. (2016). Rotator cuff tear shape characterization: A comparison of two-dimensional imaging and three-dimensional magnetic resonance reconstructions. J Shoulder Elbow Surg.

[11] Milano G., Grasso A., Salvatore M., Zarelli D., Deriu L., Fabbriciani C. (2007). Arthroscopic rotator cuff repair with and without subacromial decompression: A prospective randomized study. Arthroscopy.

[12] Nelson C.O., Sileo M.J., Grossman M.G., Serra-Hsu F. (2008). Single-row modified Mason-Allen versus double-row arthroscopic rotator cuff repair: A biomechanical and surface area comparison. Arthroscopy.

[13] Di Benedetto P., Mancuso F., Tosolini L., Buttironi M.M., Beltrame A., Causero A. (2021). Treatment options for massive rotator cuff tears: A narrative review. Acta Biomed.

[14] Hurley E.T., Maye A.B., Mullett H. (2019). Arthroscopic rotator cuff repair: A systematic review of overlapping meta-analyses. JBJS Rev.

[15] Bedeir Y.H., Schumaier A.P., Abu-Sheasha G., Grawe B.M. (2019). Type 2 retear after arthroscopic single-row, double-row and suture bridge rotator cuff repair: A systematic review. Eur J Orthop Surg Traumatol.

[16] Zhang H.-Z., Zhou Y.-F., Li W.-P. (2021). Tibiofemoral contact mechanics after horizontal or ripstop suture in inside-out and transtibial repair for meniscus radial tears in a porcine model. Arthroscopy.

[17] Bhalerao N., Pagdal S. (2019). A modified Mason-Allen technique for mini open transosseous rotator cuff repair. Int J Orthop.

[18] Lee B.G., Cho N.S., Rhee Y.G. (2012). Modified Mason-Allen suture bridge technique: A new suture bridge technique with improved tissue holding by the modified Mason-Allen stitch. Clin Orthop Surg.

